# Supplementary material for: Identification of photoperiod-induced specific miRNAs in the adrenal glands of Sunite sheep (Ovis aries)
Source: Front Vet Sci. 2022 Jul 22;9:888207. doi: 10.3389/fvets.2022.888207 (PMC9354845; doi:10.3389/fvets.2022.888207)
Supplement: Supplementary file 1 [file Data_Sheet_1.ZIP › Supplementary file/miRDeep2 file.zip!/miRDeep2 file/Know_Structure/SP42A3/oar-mir-369.pdf]

miRBase precursor : oar-mir-369
Total read count : 7861
oar-miR-369-5p read count : 554
oar-miR-369-3p read count : 7306
remaining reads : 1

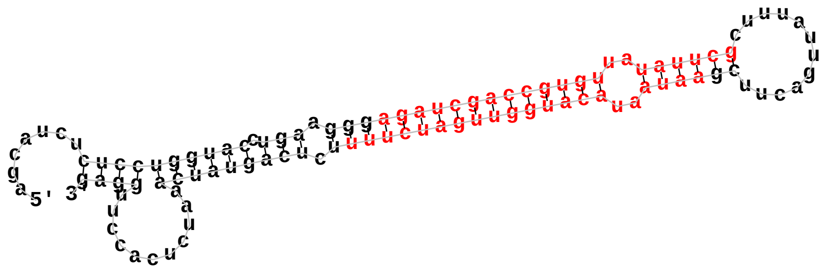

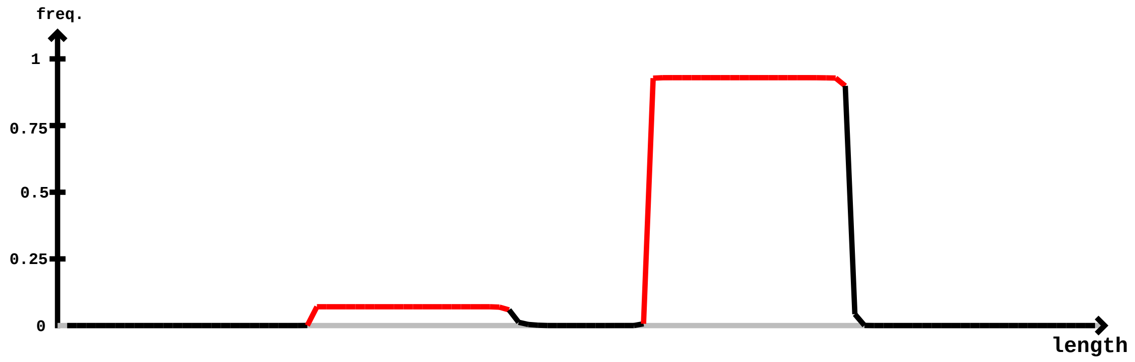

oar-miR-369-5p

oar-miR-369-3p

```
5'- agcaucucuccugguaccugaagggagaucgaccguguuauauucgcuuuauugacuucgaauaauacaugguugaucuuuucucaguaucaaaucucaccuuggag   -3'    exp
    .......((((((((((.(((.((((((((((((((((..((((((............))))))..)))))))))))))))).))))))))).............))))   reads    mm    sample
    ........................gagaucgaccguguuauauuc..............................................................   1        0     seq
    ........................gagaucgaccguguuauauucg.............................................................   2        0     seq
    .........................agaucgaccguguuauauu...............................................................   11       0     seq
    .........................agaucgaccguguuauauuc..............................................................   80       0     seq
    .........................agaucgaccguguuauauucg.............................................................   363      0     seq
    .........................agaucgaccguguuauauucgc............................................................   65       0     seq
    .........................agaucgaccguguuauauucgcu...........................................................   22       0     seq
    .........................agaucgaccguguuauauucgcuu..........................................................   9        0     seq
    .........................agaucgaccguguuauauucgcuuu.........................................................   1        0     seq
    ........................................................uucgaauaauacaugguugaucuuuu.........................   1        0     seq
    ...........................................................gaauaauacaugguugaucuu...........................   6        0     seq
    ...........................................................gaauaauacaugguugaucuuu..........................   45       0     seq
    ...........................................................gaauaauacaugguugaucuuuu.........................   1        0     seq
    ............................................................aauaauacaugguugau..............................   1        0     seq
    ............................................................aauaauacaugguugauc.............................   4        0     seq
    ............................................................aauaauacaugguugaucu............................   6        0     seq
    ............................................................aauaauacaugguugaucuu...........................   225      0     seq
    ............................................................aauaauacaugguugaucuuu..........................   6675     0     seq
    ............................................................aauaauacaugguugaucuuuu.........................   328      0     seq
    ............................................................aauaauacaugguugaucuuuuc........................   1        0     seq
    .............................................................auaauacaugguugaucuuu..........................   9        0     seq
    .............................................................auaauacaugguugaucuuuu.........................   2        0     seq
    .............................................................auaauacaugguugaucuuuuc........................   2        0     seq
    ..............................................................uaauacaugguugaucuuuuc........................   1        0     seq
```
